# Activity of Antimicrobial Peptides and Conventional Antibiotics against Superantigen Positive *Staphylococcus aureus* Isolated from the Patients with Neoplastic and Inflammatory Erythrodermia

**DOI:** 10.1155/2011/270932

**Published:** 2011-05-12

**Authors:** Wioletta Baranska-Rybak, Oscar Cirioni, Malgorzata Dawgul, Malgorzata Sokolowska-Wojdylo, Lukasz Naumiuk, Aneta Szczerkowska-Dobosz, Roman Nowicki, Jadwiga Roszkiewicz, Wojciech Kamysz

**Affiliations:** ^1^Department of Dermatology, Venereology and Allergology, Medical University of Gdansk, 80-210 Gdansk, Poland; ^2^Institute of Infectious Diseases and Public Health, Marche Polytechnic University, 60121 Ancona, Italy; ^3^Department of Inorganic Chemistry, Faculty of Pharmacy, Medical University of Gdansk, Al. Gen. J. Hallera 107, 80-416, Gdansk, Poland

## Abstract

Superantigens are proteins comprising a group of molecules produced by various microorganisms. They are involved in pathogenesis of several human diseases. The aim of the study was the comparison of susceptibility to antibiotics and antimicrobial peptides (AMPs) of *Staphylococcus aureus* (SA) strains producing staphylococcal enterotoxins SEA, SEB, SEC, SED, and TSST-1 and nonproducing ones. In the group of the total 28 of the patients with erythrodermia the presence of SA was confirmed in 24 cases. The total of 14 strains of SA excreted enterotoxins SEA, SEC, SED, and TSST-1. We did not observe that strains producing mentioned superantigens were less susceptible to AMPs (aurein 1.2, citropin 1.1, lipopeptide, protegrin 1, tachyplesin 3, temporin A, and uperin 3.6). The opposite situation was observed in conventional antibiotics. SA strains excreting tested superantigens had higher MICs and MBCs than nonproducing ones. The interesting finding considering the high efficacy of AMPs, against all examined strains of SA, makes them attractive candidates for therapeutic implication.

## 1. Introduction

Superantigens are proteins comprising a group of molecules produced by various microorganisms, such as bacteria (staphylococci, streptococci, and mycoplasma), fungi (yeasts), and viruses. They are involved in the pathogenesis of several human diseases (atopic eczema, toxic shock syndrome, psoriasis, and Kawasaki disease) [1]. Superantigens are characterized by their capacity to stimulate a large number of T-cells. In contrast to conventional antigens, superantigens bypass avoid intracellular processing and bind directly to the major histocompatibility complex (MHC) class II molecule, on the surface of the antigen processing cell, outside the antigen-binding groove [2]. T-cells belonging to both the CD4 and CD8 subtype are activated. T-cell activation in the presence of superantigens may lead to the activation of several percent of the total T-cell population, and thereby activate by a factor of more than 10–100 the number of T-cells activated in the presence of conventional antigens [3]. 

 Some 80 to 100 percent of atopic dermatitis (AD) patients have skin colonization with *Staphylococcus aureus* (SA) [4]. It has been found on both the healthy and lesional skin of those patients. SA superantigens are a well-known AD-exacerbating factor. The pathogens concentration (cfu/cm^2^) on the skin of atopic dermatitis patients is significantly higher than on that of healthy population [5]. Suppressed levels of ceramides, free lipoid acids, superficial polar lipids, skin natural antimicrobial peptides (LL-37, *β*-defensin), as well as the pH shifted to alkaline region (7-8), fibronectin receptors exposure of adhesin-binding cell wall of SA, and destruction of the skin barrier by substances excreted by these germs are responsible for SA skin colonization in AD [6–8]. 

The aggravating role of SA superantigens is well known in AD but has not been well documented in psoriasis. There are single reports concerning correlation between the severity of AD and psoriasis and enterotoxin production of isolated SA strains [9].

 There are single reports confirming the relationship between erythrodermic cutaneous T-cell lymphoma (CTCL) and superantigens producing SA colonization [10]. It has been demonstrated that antibiotic therapy in CTCL can suppress inflammation and the size of neoplastic tumours in those cases. The relationship between staphylococcal skin infections and erythrodermic CTCL needs exploration. It can be hypothesized that like other T-cell-mediated skin diseases, CTCL occurs in the setting of a genetically determined host (HLA determinants), a trigger (antigens or superantigens), and an immune response with cytokine and chemokine production. In CTCL, T-cells are attracted into the epidermis, and they may be unable to limit their proliferation (absent apoptosis). SA superantigen presented either by Langerhans cells or by class-II-bearing keratinocytes results in cytokines that stimulate T-cells. It is reasonable that persistent colonization with toxigenic bacteria would expand the population of epidermotropic T-cells and elicit production of T-cell-activating cytokine/chemokines [11, 12]. 

Bering in mind that the enhanced resistance of bacteria to conventional antibiotics is a serious problem in present-day healthcare, the development of novel antimicrobial therapies, such as those based on various antimicrobial peptides, seems to be reasonable. 

Humans express a blend of antimicrobial peptides (AMPs), which are found at biological boundaries prone to infection. One of the most important innate defense mechanisms of the human skin is the production of AMPs. They are produced mainly by keratinocytes in the stratum corneum, neutrophils, or by sweat glands and are either expressed constitutively like RNase 7, psoriasin, or dermcidin or after an inflammatory stimulus like *β*-defensin-2 (HBD-2) and -3 (HBD-3) or the cathelicidin LL-37 [13]. AMPs kill bacteria by permeating their membranes, and thus, the lack of a specific molecular microbial target minimizes resistance development [14]. Actually, several peptides and peptide-based compounds are passing clinical trials [15]. Expression levels of these natural antibiotics correlate well with susceptibility to skin infections [16]. 

Herein, we investigated SA colonization in patients with erythrodermia (a skin inflammation of more than 90% of body surface) that developed in the course of psoriasis, atopic dermatitis, cutaneous T-cell lymphoma, or Sezary Syndrome (SS) [17, 18]. The isolated bacterial strains were analyzed for the superantigen excretion and susceptibility to conventional antibiotics and selected AMPs (aurein 1.2, citropin 1.1, lipopeptide Pal-KK-NH_2_, protegrin 1, tachyplesin 3, temporin A, and uperin 3.6).

## 2. Materials and Methods

### 2.1. Antimicrobial Peptides

Peptides (aurein 1.2, citropin 1.1, lipopeptide Pal-KK-NH_2_, protegrin 1, tachyplesin 3, temporin A, and uperin 3.6) included in the study were synthesized manually in a microwave reactor by the solid-phase method using the 9-fluorenylmethoxycarbonyl chemistry (Fmoc) [19, 20]. The completeness of each coupling reaction was monitored by the chloranil test. The peptides were cleaved from the solid support by trifluoroacetic acid (TFA) in the presence of water (2.5%) and triisopropylsilane (2.5%) as scavengers. The cleaved peptides were precipitated with diethyl ether, and cysteine-containing ones peptides were oxidized by 0.1 M iodine in methanol. The peptides were purified by high-performance liquid chromatography (HPLC). The resulting fractions of purity greater than 95%–98% were tested by HPLC and thin layer chromatography (TLC) for lipopeptide. The peptides were analyzed also by matrix-assisted laser desorption ionization time of flight mass spectrometry (MALDI-TOF MS).

### 2.2. Bacterial Isolates and Antibiotics Disk Susceptibility Test

Twenty-eight patients with erythrodermia, hospitalized at the Department of Dermatology, Venereology, and Allergology from January 2007 to October 2008, were enrolled in the study. From each patient, skin swabs were taken. All samples were cultured on the Columbia agar plates (*Becton Dickinson, Germany*) using standard microbiological procedures. SA was identified on the basis of colony morphology, production of catalase, and positive slide coagulation test (*Staphaurex *and* Biomerieux*). The susceptibility to antibiotics was determined by disk diffusion method as recommended by CLSI (Clinical Laboratory Standards Institute) guidelines. The following antimicrobials were tested: penicillin, oxacillin, erythromycin, doxycycline, clindamycin, rifampicin, chloramphenicol, linezolid, daptomycin, tigecycline, and ciprofloxacin (*Oxoid, UK*). The susceptibility tests were performed on the Mueller-Hinton II (*Becton Dickinson*). The results obtained by disk diffusion were compared to those of the broth microdilution in the case of linezolid, daptomycin, tigecycline, rifampicin, chloramphenicol, erythromycin, and clindamycin.

### 2.3. Enterotoxin Detection

A staphylococcal enterotoxin test kit (*SET-RPLA KIT TOXIN DETECTION KIT, Oxoid*) was used for the detection of staphylococcal enterotoxins A, B, C, and D in culture by reversed passive latex agglutination. Clinical strains of SA were incubated in Tryptone Soy Broth (*Becton Dickinson*) and incubated at 37°C for 18–24 hours, with shaking on a water bath. After growth, they were centrifuged at 900 g for 20 minutes at 4°C, and the supernatants were used as the test sample. Latex sensitised with antienterotoxin A, B, C, or D was added to filter-sterilized supernatants on V-well microtiter plates (*Sigma-Aldrich, Germany*). A visible agglutination on the bottom of the well was considered as a positive result.

### 2.4. TSST Detection

A staphylococcal toxic shock syndrome toxin kit (*TST-RPLA TOXIN DETECTION KIT, Oxoid*) was used for the detection of a staphylococcal toxic shock syndrome toxin in culture by reversed passive latex agglutination. Clinical strains of *S. aureus* were *incubated* in a brain-heart infusion broth (*Becton Dickinson*) and incubated at 37°C for 18–24 hours, with shaking on a water bath. After growth, they were centrifuged at 900 g for 20 minutes at 4°C, and the supernatants were used as the test sample. Latex sensitized with antitoxin was added to filter-sterilized supernatants on V-well microtiter plates (*Sigma-Aldrich*). A visible agglutination on the bottom of the well was considered as a positive result.

### 2.5. Microorganisms and Antimicrobial Assay

A total number of 24 SA strains were obtained from patients with erythrodermia and 3 reference SA ones from the American Type Culture Collection: 6538P ATCC, 9144 ATCC, and 25923 ATCC (*Institute of Experimental Therapy, Wroclaw, Poland*). MIC was determined using a microbroth dilution method with the Mueller Hinton Broth II (MHB II) (*Becton Dickinson*) and initial inoculums of 5 × 10^5^ CFU/mL. Polypropylene 96-well plates (*Sigma-Aldrich*) were incubated for 18 h at 37°C. MIC was taken as the lowest drug concentration at which a noticeable growth was inhibited. MBC was taken as the lowest concentration of each drug that resulted in more than 99.9% reduction of the initial inoculums. The experiments were performed in triplicate.

## 3. Results

### 3.1. Staphylococcus Aureus Isolation

In a group of 28 patients with erythrodermia (11 in the course of psoriasis, 9 with atopic dermatitis, 6 with CTCL, and 2—Sezary syndrome) the presence of SA was confirmed in 24 cases. Negative cultures for SA were noticed in three patients with psoriasis.

### 3.2. Superantigens Detection

A total number of 14 SA strains excreted enterotoxins SEA (8 strains), SEC (3 strains), and/or SED (5 strains) and only one TSST-1 in the group of 24. Intermediate results (+/−) were considered as negative. In the group of 9 patients with AD, the superantigen-producing strains were detected in 6 patients, in the group of 6 patients with CTCL—in 3 cases and in 8 patients with psoriasis—in 3 cases. SA strains isolated from two patients with SS did not produce the above-mentioned superantigens.

### 3.3. Antibiotics and Antimicrobial Peptides Susceptibility

The susceptibility to antibiotics determined by the disk diffusion method and broth microdilution gave identical results (data not shown). The antibiotics used in the study, rifampicin, tigecyline, vancomycin, daptomycin, ciprofloxacin, chloramphenicol, clindamycin, and erythromycin, exhibited diverse activities against clinical isolates of SA. The rifampicin, tigecycline, vancomycin, and daptomycin MICs values, which were the lowest among the tested antibiotics, varied between 1 and 4 mg/L. The other ones were higher than the tested antimicrobial peptides: tachyplesin 3, lipopeptide, and protegrin 1 were extremely effective against all the tested bacterial strains (MIC values between 1 and 8 mg/L); see Table 1. The reference strains were more susceptible to both conventional antibiotics and AMPs than the clinical ones; see Table 2.

### 3.4. Correlation Study

**Figure 1:**
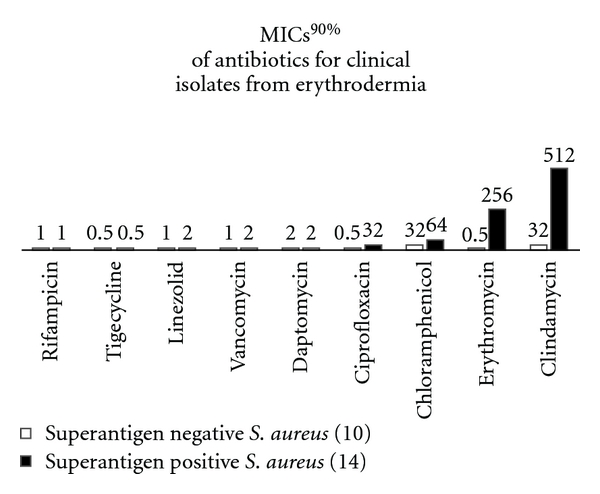
The relationship between superantigen production and susceptibility to conventional antibiotics.

**Figure 2:**
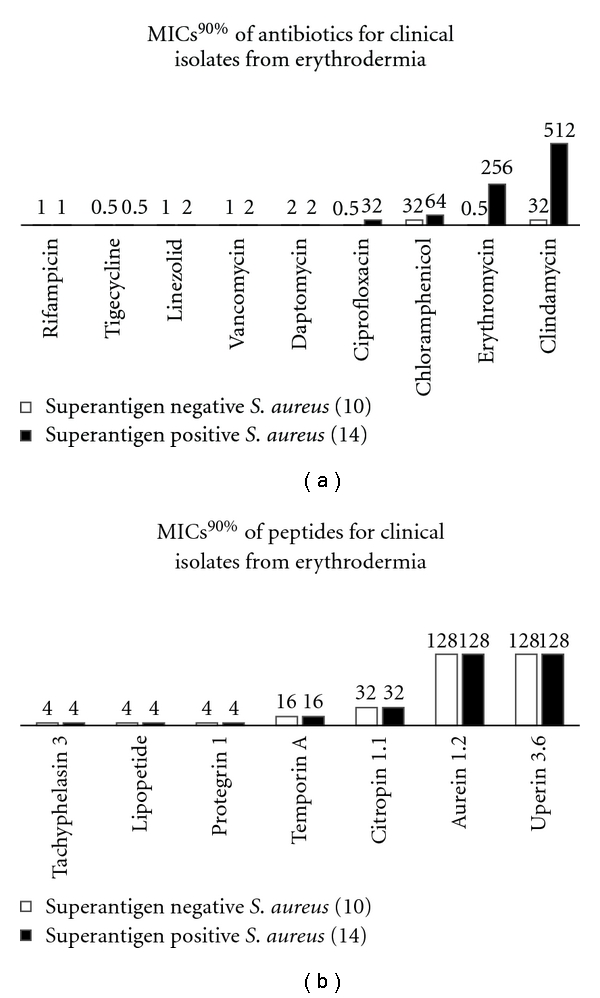
The relationship between superantigen production and susceptibility to antimicrobial peptides.

We did not notice that strains producing tested superantigens (SEA, SEC, SED, and TSST-1) were less susceptible to AMPs than nonproducing ones. The opposite situation was observed in conventional antibiotics. SA strains excreting those superantigens had higher MICs and MBCs Figures 1 and 2.

## 4. Discussion

Bacterial superantigens, which stimulate clonal expansion of T-cells by mechanisms involving specific HLA molecules, have also been hypothesized to cause inflammatory skin diseases [10]. The mechanisms by which these toxins act remain still unknown. This is the first report of the occurrence of staphylococcus superantigens in erythrodermic skin diseases (AD, psoriasis, CTCL, and SS). 

There are many studies that explain the effect of SA on AD [21]. Most SA strains isolated from AD patients can produce superantigenic toxins such as staphylococcal enterotoxin SEA, SEB, SEC, SED, and the toxic shock syndrome toxin-1 (TSST-1) that correspond well with our findings (66.7% of strains excreted tested superantigens). Colonization and infection with *Staphylococcus *and *Streptococcus *have been reported to exacerbate psoriasis [22, 23]. The presence of SA in psoriatic erythrodermia was confirmed in 8 out of 11 patients, while the ability to produce examined superantigens was detected in 3 strains. CTCL patients resemble those with acquired immunodeficiency syndrome who cannot clear the skin off staphylococcus and have protracted pruritus and erythrodermic psoriasis [10]. The association between staphylococcal colonization and the erythrodermic form of CTCL deserves further attention and study. The strains excreting specified superantigens colonized 50% of patients with CTCL in our study.

We found that 24 out of 28 erythrodermic patients had a staphylococcal culture positive from the skin, and tested superantigens were detected in SA strains isolated from 14 patients. The purpose of our study was to investigate whether or not the strains producing SEA, SEC, SED, and TSST-1 are more resistant to conventional antibiotics and AMPs. Considering susceptibility to antimicrobial peptides, we did not notice any significant differences between strains producing tested superantigens and nonproducing strains. The opposite situation was noticed in susceptibility to conventional antibiotics. The SA strains producing specified superantigens had higher MICs and MBCs as compared to the nonproducing ones. Especially alarming is the higher resistance of those strains to macrolides and lincosamides which could not only kill bacteria and diminish the rate of colonization but also inhibit their superantigen and toxin production [24, 25]. One study showed that *β*-lactams which target cell wall development in bacteria and are the basis for the treatment of skin and soft-tissue infections could even increase the production of toxins [25]. SA strains which can produce superantigens and toxins and additionally acquire the mechanism (i.e., resistance) protecting their production are the most difficult to control. Adachi et al. speculated that inhibitors of protein synthesis may have an antimicrobial effect and also inhibitory effect on superantigen production from SA [24]. In fact, the inhibition of superantigen production by antibiotics may not be sufficient to justify clinical efficacy.

Over 50% incidence of production of tested superantigens in strains from AD patients is in accordance with previous studies [26].

Several studies on the effect of antimicrobial treatment on the colonization with SA and the severity of inflammation gave conflicting results. In several open or double-blind placebo-controlled trials, topical or systemic antibiotics were able to reduce colonization density and led to a partial improvement of skin lesions [27–29]. On the other hand, treatment with oral antibiotics did not lead to a significant improvement of AD in two double-blind placebo-controlled studies [30, 31]. No matter what kind of the treatment has been adopted, recolonization occurred after 4–8 weeks [32].

## 5. Conclusions

From a clinical point of view, our study has several implications. Considering that erythrodermic patients are frequently treated with various antibiotics, the question may be raised whether excessive use of antibiotics and induction of resistance are associated with cross-resistance to AMPs. We found no evidence for the development of the AMPs resistance in relation to antibiotic susceptibility, likely reflecting the fact that the mode of action of the antibiotics investigated herein is not shared with AMPs. An interesting finding of the high efficacy of AMPs, especially lipopeptides, against all tested strains of SA makes them attractive candidates for therapeutic application.

##  Conflict of Interests

The authors declare that they have no conflict of interests.

## Figures and Tables

**Table 1:** The activity of antimicrobial peptides and conventional antibiotics against *S. aureus *clinical isolates.

Strain (no. of isolates) and agent	MIC (mg/liter)			MBC (mg/liter)		
	Range	50%	90%	Range	50%	90%
*Superantigen negative S. aureus* (10)						
*Tachyplesin 3 *	1–4	2	4	2–4	2	4
*Lipopeptide *	1–8	2	4	2–8	4	8
*Protegrin 1 *	1–4	2	4	2–8	4	8
*Temporin A *	8–32	8	16	16–64	16	32
*Citropin 1.1 *	8–32	16	32	16–64	16	64
*Aurein 1.2 *	32–128	64	128	64–128	64	128
*Uperin 3.6 *	128–256	128	128	128-128	128	128
*Rifampicin *	0.25–1	0.25	1	1-2	0.25	1
*Tigecycline *	0.5–1	0.5	0.5	1-2	1	1
*Linezolid *	0.5–2	1	1	NT	NT	NT
*Vancomycin *	0.5–1	1	1	1–4	1	2
*Daptomycin *	1–4	1	2	1–4	2	2
*Ciprofloxacin *	0.25–0.5	0.25	0.5	1–8	1	4
*Chloramphenicol *	2–32	4	32	8–32	8	32
*Erythromycin *	0.25–64	0.5	0.5	1–4	1	2
*Clindamycin *	16–64	32	32	64–128	32	64
*Superantigen positive S. aureus* (14)						
*Tachyplesin 3 *	1–4	2	4	2–4	2	4
*Lipopeptide *	1–8	2	4	2–8	4	8
*Protegrin 1 *	1–4	2	4	2–8	4	8
*Temporin A *	8–32	8	16	16–64	32	64
*Citropin 1.1 *	8–32	16	32	16–64	16	64
*Aurein 1.2 *	32–128	64	128	64–128	64	128
*Uperin 3.6 *	128–256	128	128	128-128	128	128
*Rifampicin *	0.25–1	0.25	1	0.5–2	1	2
*Tigecycline *	0.5–1	0.5	0.5	1-2	1	1
*Linezolid *	0.5–2	1	2	NT	NT	NT
*Vancomycin *	0.5–2	1	2	1–4	2	4
*Daptomycin *	1–4	1	2	1–4	2	4
*Ciprofloxacin *	0.25–128	2	32	2–128	4	64
*Chloramphenicol *	4–128	8	64	8–128	16	128
*Erythromycin *	0.25–512	1	256	4–512	8	128
*Clindamycin *	8–512	64	512	16–512	64	128

**Table 2:** The activity of antimicrobial peptides and conventional antibiotics against *S. aureus* referential strains.

		MIC (mg/liter)	
	ATCC 6538P	ATCC 9144	ATCC 25923
*Tachyplesin 3*	2	2	2
*Lipopeptide*	2	2	4
*Protegrin 1*	4	2	4
*Temporin A*	8	16	16
*Citropin 1.1*	8	32	32
*Aurein 1.2*	64	64	64
*Uperin 3.6*	64	64	128
*Rifampicin*	0.25	0.25	0.25
*Tigecycline*	0.25	0.25	0.25
*Linezolid*	0.5	1	0.5
*Vancomycin*	1	2	1
*Daptomycin*	2	2	2
*Ciprofloxacin*	1	2	1
*Chloramphenicol*	4	4	4
*Erythromycin*	1	1	1
*Clindamycin*	4	2	2

## Abbreviations

SA: *Staphylococcus aureus*
SEA: Staphylococcal enterotoxin A
SEB: Staphylococcal enterotoxin B
SEC: Staphylococcal enterotoxin C
SED: Staphylococcal enterotoxin D
TSST: Staphylococcal toxic shock syndrome toxin
AMPs: Antimicrobial peptides
AD: Atopic dermatitis
CTCL: Cutaneous T-cell lymphoma
SS: Sezary syndrome
MIC: Minimal inhibitory concentration
MBC: Minimal bactericidal concentration.
